# PyMYB10 and PyMYB10.1 Interact with bHLH to Enhance Anthocyanin Accumulation in Pears

**DOI:** 10.1371/journal.pone.0142112

**Published:** 2015-11-04

**Authors:** Shouqian Feng, Shasha Sun, Xiaoliu Chen, Shujing Wu, Deyun Wang, Xuesen Chen

**Affiliations:** State Key Laboratory of Crop Biology, College of Horticulture Science and Engineering, Shandong Agricultural University, Tai-An, Shandong, China; Shanghai Institutes for Biological Sciences, CHINA

## Abstract

Color is an important agronomic trait of pears, and the anthocyanin content of fruit is immensely significant for pear coloring. In this study, an anthocyanin-activating R2R3-MYB transcription factor gene, *PyMYB10*.*1*, was isolated from fruits of red sand pear (*Pyrus pyrifolia* cv. Aoguan). Alignments of the nucleotide and amino acid sequences suggested that PyMYB10.1 was involved in anthocyanin regulation. Similar to *PyMYB10*, *PyMYB10*.*1* was predominantly expressed in red tissues, including the skin, leaf and flower, but it was minimally expressed in non-red fruit flesh. The expression of this gene could be induced by light. Dual-luciferase assays indicated that both PyMYB10 and PyMYB10.1 activated the *AtDFR* promoter. The activation of *AtDFR* increased to a greater extent when combined with a bHLH co-factor, such as PybHLH, MrbHLH1, MrbHLH2, or AtbHLH2. However, the response of this activation depended on the protein complex formed. PyMYB10-AtbHLH2 activated the *AtDFR* promoter to a greater extent than other combinations of proteins. PyMYB10-AtbHLH2 also induced the highest anthocyanin accumulation in tobacco transient-expression assays. Moreover, PybHLH interacted with PyMYB10 and PyMYB10.1. These results suggest that both PyMYB10 and PyMYB10.1 are positive anthocyanin biosynthesis regulators in pears that act via the formation of a ternary complex with PybHLH. The functional characterization of *PyMYB10* and *PyMYB10*.*1* will aid further understanding of the anthocyanin regulation in pears.

## Introduction

Pear is an economically important temperate fruit. Until to now, at least 22 primary species of *Pyrus* have been identified; however, only the four major species *Pyrus bretschneideri*, *Pyrus pyrifolia*, *Pyrus ussuriensis*, and *Pyrus communis* have been utilized for commercial fruit production [1].The sand pear (*P*. *pyrifolia*)is primarily cultivated in eastern Asia. In general, the sand pear can be divided into four types based on skin color: red, green, russet, and an intermediate color (russet and green). Most cultivated sand pear varieties are green and russet. Although red pears exist, their supply is inadequate [2].Thus, red color has recently become an important breeding objective for pear cultivars, especially in Asian countries such as China.

The red coloration of pears is mainly determined by the skin anthocyanin content [3]. Cyanidin-3-galactosideand peonidin-3-galactoside are the main anthocyanin components of pear skin[4, 5]. The anthocyanin biosynthetic pathway of pears is usually divided into two main sections: chalcone synthase (CHS), chalconeisomerase (CHI) and flavanone 3-hydroxylase (F3H) are in the early section, dihydroflavonol 4-reductase(DFR), anthocyanidin synthase (ANS) and UDP-glucose: flavonoid 3-O-glucosyltransferase (UFGT) are in the late section. Genes of these enzymes have been well characterized and they are primarily regulated by transcription factors at the transcriptional level [6, 7, 8].

MYB and basic helix loop helix (bHLH) transcription factor have been commonly identified as anthocyanin regulators in model plants and fruits, including grapes [9], apples [10], and Chinese bayberries [11]. Notably, MYB appears to play a key role in anthocyanin accumulation. MYB proteins, the largest transcription factor family in plants, are identified based on the number of the MYB conserved domain (R1-MYB, R2R3-MYB, or R1R2R3-MYB). Most MYB TFs involved in the regulation of anthocyanin pathway are R2R3-MYB TFs. With respect to their distinct functions, they are usually divided into two groups. One group consists of most known positive anthocyanin regulators, including *PhAN2* and *PhAN4* in petunias [12], *PAP1* and *PAP2* in *Arabidopsis* [13], *MdMYB10* in apples [10], and *VvMYBA1* in grapes [14].The members of the second group were served as anthocyanin repressors, including *FaMYB1* in strawberry [15]; *AtMYB6*, *AtMYB4*,and *AtMYB3* in *Arabidopsis* [16, 17, 18]; and *AmMYB308* in *Antirrhinum* [19].Many researchers have shown that changes to these R2R3-MYB TFs can markedly affect phenotype. For example, red-fleshed apples are the result of a tandem repeats in the *MdMYB10* promoter [20].Ectopic expression assays suggested that R2R3-MYB can work independently or together with bHLH in controlling anthocyanin biosynthesis. For example, the maize P factor has been demonstrated to activate a subset of anthocyanin biosynthetic genes independently of a bHLH coactivator [21].Conversely, the maize C1 factor has been shown to cooperate with R, to activate the promoter of *DFR* [22]. Based on the theoretical progress made in petunia, apple and *Arabidopsis*, the first R2R3-MYB TF in sand pear, *PyMYB10*, was isolated and reported to regulate anthocyanin synthesis in red-skinned pears [23]. However, the interactions between PyMYB10 and bHLH proteins are not well understood.

In the present study, a R2R3-MYB, *PyMYB10*.*1*, was isolated from the red sand pear (cv. Aoguan). PyMYB10.1 shares a high level of sequence homology with other known anthocyanin regulators. Phylogenetic results revealed that PyMYB10.1 and PyMYB10 are in the same clade. In addition, *PyMYB10*.*1* and *PyMYB10* are preferentially expressed in tissues where anthocyanin accumulates. A dual-luciferase assay indicatedthatPyMYB10.1 and PyMYB10could activate the *AtDFR* promoter in the presence of a bHLH co-factor. Furthermore, yeast two-hybrid and BiFC assays confirmed that PyMYB10.1 and PyMYB10 interact with PybHLH TF. Lastly, the in vitro transient expression of PyMYB10 and PyMYB10.1 induced differential accumulation of anthocyanin in the injection area of tobacco leaves when co-expressed with several bHLH TFs.

## Materials and Methods

### Plant materials

No specific permit was required for this experiment. The location is not protected in any way, and the study did not involve endangered or protected species.

All the tissues were collected from ‘Aoguan’ plants. Plants were grown at Aoguan FruitCorp. (lat: 116.2501, lng: 36.589; elevation: 31m). The skins were collected from ‘Aoguan’ on September 2012, 6 days after debagging. The samples were immediately frozen by liquid nitrogen, and then stored at -80°C for subsequent experiments.

### Anthocyanin content analysis

Anthocyanins were extracted according to the method described by Pirie and Mullins [24] and Wang et al. [25]. Absorbance of the extracts was monitored at 553 and 600 nm. Anthocyanin content was calculated as described by Wang et al. [25]. Three replicates of each sample were analyzed.

### Total RNA extraction and cDNA synthesis

Total RNA was extracted according to a modified cetyltrimethylammoniumbromide (CTAB) method [26], and then treated by DNase I (Fermentas, USA). cDNA synthesis was performed using the Revert Aid™ First Strand cDNA Synthesis kit (Fermentas, USA).

### Isolation of *PyMYB10*.*1*

The *PyMYB10*.*1* gene (accession numberKT748756) was cloned from ‘Aoguan’ cDNA using degenerate primers [27].Based on homology with the R2R3-MYBs related to anthocyanin biosynthesis in other species, *PyMYB10*.*1* was selected for further study. A full-length cDNA of the *PyMYB10*.*1* was subsequently obtained by 5’-RACE with the primer 5′-TCTTCCAGCAATTATTGACCACCTG-3′ and 3’-RACE with the primer 5′-GCAGGAAAAGCTGCAGACAGAGGTG-3′ using SMART™ RACE cDNA Amplification Kit (Invitrogen, USA).

### Bioinformatic analysis

The phylogenetic tree was constructed utilized MEGA version 3.1 [28]. Multiple sequence alignments was performed with the Clustal W 2 (http://www.ebi.ac.uk/Tools/clustalw2/). The motif sequence and protein domains were identified using InterPro software.

### Real-time quantitative PCR

qPCR DNA amplification was performed using the Light Cycler System (Bio-Rad, USA). All reactions were carried out in triplicate using a volume of 20μ Lreaction mixture containing 2μL of Master Mix (TaKaRa), 0.5 M of each primer, 2μl of diluted cDNA. The qPCR reaction programs were as follows: 95°C for 5 min; 40 cycles for 10 s at 95°C, 30 s at 56°C and 30 s at 72°C; and a final extension at 72°C for 3 min. The primers used for *PyMYB10*.*1*, *PyMYB10* and *PybHLH* are listed in S1 Table. *PyActin* (accession number CN938023) was used as a constitutive control gene.

### Tobacco transient-expression assay

The promoter of *ArabidopsisDFR* (TT3, AT5g42800) was subcloned into the vector pGreenII 0800-LUC [29]. The full-length CDS of the TFs *PyMYB10*,*PyMYB10*.*1*, *MrbHLH1*,*MrbHLH2* and *AtbHLH2* were subcloned into the vector pGreenII 0029 62-SK.The tobacco (*N*. *tabacum*) abaxial leaf surface was infiltrated with *PyMYB10*, *PyMYB10*.*1*, *MrbHLH1*, *MrbHLH2*and *AtbHLH2*either singly or in pairs. Tobacco (*N*. *tabacum*) was transformed with *Agrobacterium*. The TF-promoter interactions were measured based on the ratio of LUC activity to REN activity. Three days after the transformation, the LUC and REN activities were analyzed as described by Liu et al. [11]. Digital photos of infiltration area were taken 8 days after infiltration. The primers used for full-length TF amplification are listed in S2 Table. All the statistical analyses were performed using SPSS software.

### Yeast two-hybrid assay (Y2H)

The *PyMYB10* (GU253310) and *PyMYB10*.*1*ORFs were inserted into the pGADT7 vector, and the *PybHLH* (HM622265) ORF was inserted into the pGBKT7 vectors (BD Biosciences) [30]. These clones were then used to study the PyMYB10, PyMYB10.1and PybHLH interactions in the Y2H assay. The interactions of these proteins were detected using Matchmaker yeast two-hybrid system (BD Biosciences, USA). AH109 competent cells were co-transformed with the PyMYB10, PyMYB10.1 and PybHLH constructs. The co-transformants were initially selected on synthetic dropout medium lacking Leu and Trp (SD/−Leu/−Trp) and then streaked on quadruple-dropout medium deficient in Ade, His, Leu and Trp (SD/−Ade/−His/−Leu/−Trp). X-gal was further used to assess theβ-galactosidase activity to confirm positive interactions. The primers used to construct the plasmids are presented in S3 Table.

### Bimolecular fluorescence complementation assay (BiFC)

The full-length CDS of *PyMYB10* and *PyMYB10*.*1*or *PybHLH* were cloned into the binary YFP BiFC vectors35S-pSPYCE-YFPor35S-pSPYNE-YFP, respectively, resulting in the recombinant plasmids PyMYB10-YFPC, PyMYB10.1-YFPC and PybHLH-YFPN. The primers used to construct the plasmids are presented in S4 Table. Onion epidermis cells were transformed with *Agrobacterium* as described by Li et al. [31]. Two days after transformation, the YFP signals were examined in the transfected cells using a confocal microscope (Deerfield, IL, USA).

## Results

### Isolation of PyMYB10.1 from pears

**Fig 1:**
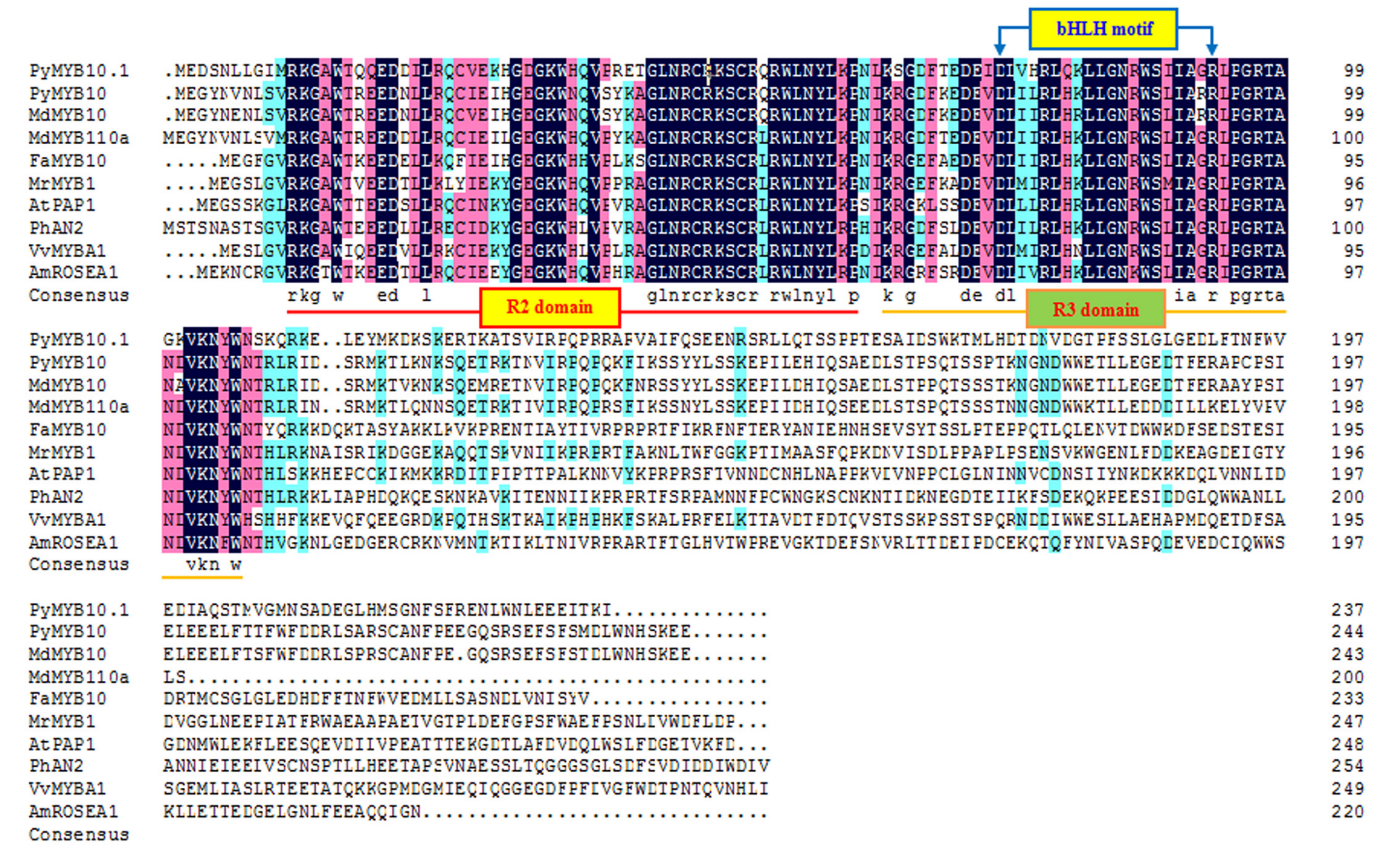
Multiple alignments of PyMYB10.1 and anthocyanin R2R3-MYB regulators. Identical amino acids are shaded in black and similar amino acids in pink or turquoise. The R2- and R3-MYB DNA-binding domains of selected MYB proteins are underlined. The bHLH binding motif is indicated with brackets. The GenBank accession numbers of R2R3-MYB proteins are as follows: *Pyrus pyrifolia* PyMYB10 (ADN52330); *Malus x domestica* MdMYB10(ACQ45201) andMdMYB110a (AB743999); *Fragaria x ananassa* FaMYB10(ABX79947); *Petunia x hybrida* PhAN2(AAF66727); *Arabidopsis thaliana* AtPAP1(AAG42001); *Vitis vinifera* VvMYBA1(BAD18977); and *Antirrhinum majus* AmROSEA1(ABB83826).

Using degenerate primers [27], a 249-bp R2R3-MYB fragment was isolated from ‘Aoguan’ cDNA samples. Then the 714-bp full-length ORF cDNA was obtained by RACE PCR. The predicted protein is 237 amino acids in length with a calculated molecular mass of 27.4kDa and an isoelectric point of 8.78.An alignment of the deduced amino acid sequence with known anthocyanin MYB regulators indicated high conserved at the R2R3-MYB domain particularly with PyMYB10, but the C-termini downstream of this region are more divergent (Fig 1).The R3-MYBdomain of the conserved N-terminal portion of the protein sequence contains the bHLH-binding region motif ([DE]Lx_2_[RK]x_3_Lx_6_Lx_3_R). At the protein level, the predicted amino acid sequence has 51%homology with PyMYB10 from pears, 49% homology with MdMYB10 from apples, and 44% homology with VvMYBA1 from grapes. It suggested that a new homologous gene of *PyMYB10* was isolated from red sand pear, and we named this sequence *PyMYB10*.*1*.

**Fig 2:**
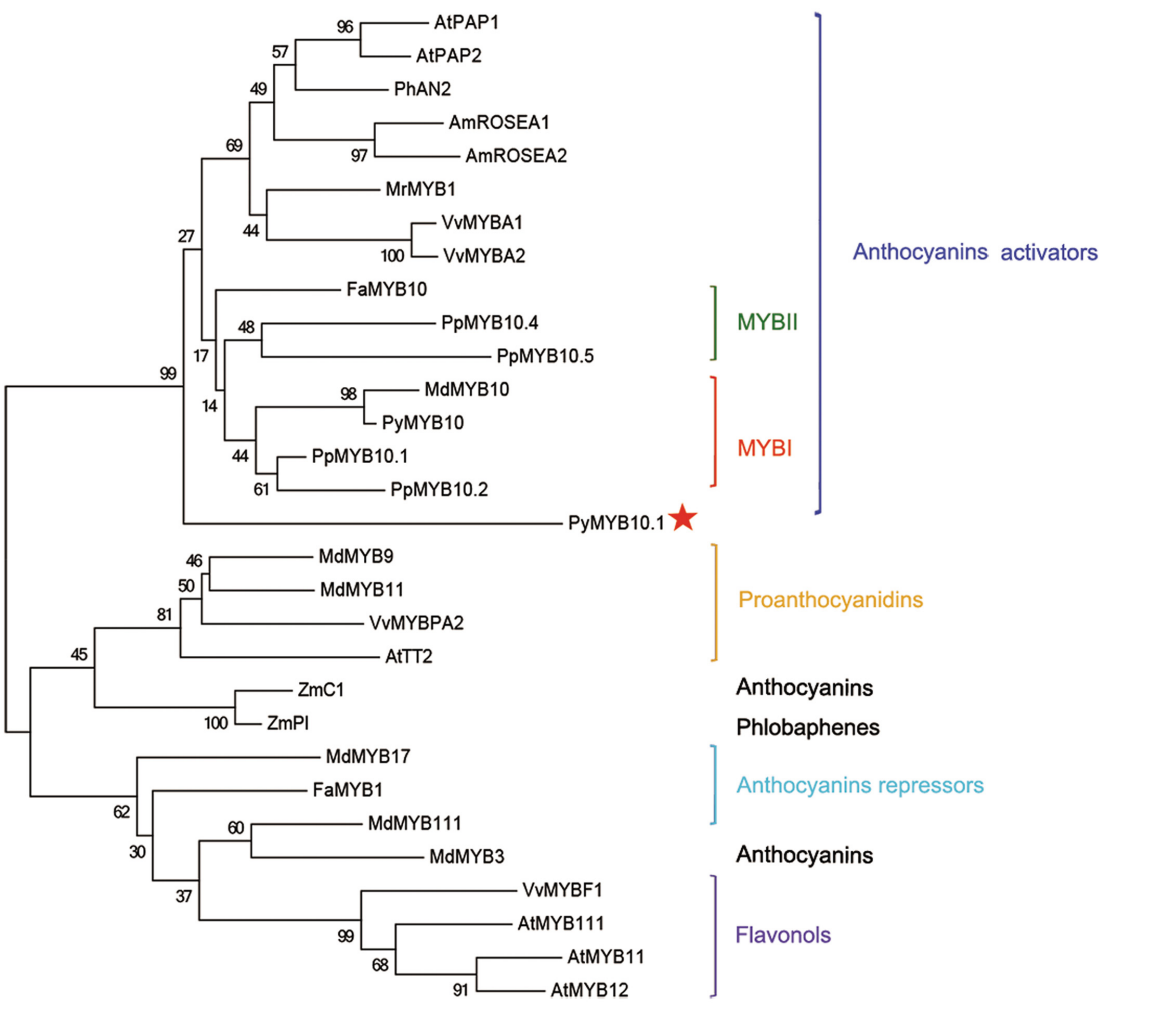
Phylogenetic relationship of PyMYB10.1 to other R2R3-MYBs. A phylogenetic tree was constructed using the neighbor-joining method by the MEGA3 software. The reliability of the trees was tested using a bootstrapping method with 1000 replicates. PyMYB10.1 is indicated with an asterisk. Putative regulatory functions of the selected R2R3-MYB proteins are indicated. The GenBank accession numbers of some R2R3-MYB protein sequences are as follows: *Antirrhinum majus* AmROSEA2 (ABB83827); *Prunus persica* PpMYB10.1 (Ppa026640m), PpMYB10.2 (Ppa016711m), PpMYB10.4 (Ppa018744m), PpMYB10.5(Ppa024617m); *Arabidopsis thaliana* AtMYB11 (EFH52939), AtMYB12 (AEC10843), AtMYB111 (EFH41988), AtPAP2 (AAG42002), and AtTT2 (AED93980); *Fragaria ananassa* FaMYB1 (AAK84064); *Malus domestica*MdMYB3 (AEX08668.1), MdMYB9 (ABB84757), MdMYB11 (AAZ20431), MdMYB17 (ADL36757), and MdMYB111 (ADL36754); *Pyrus pyrifolia* PyMYB10 (ADN52330); *Morella rubra* MrMYB1 (ADG21957); *Zea mays* ZmC1 (AAA33482),ZmPl (AAA19819);and *Vitis vinifera* VvMYBA2 (BAD18978), VvMYBF1 (ACV81697), and VvMYBPA2 (ACK56131).

To further analyze the relationship between PyMYB10.1 and other R2R3-MYB transcription factors, a phylogenetic tree was constructed. A phylogenetic analysis revealed that PyMYB10.1 clusters with the pear anthocyanin MYB regulator, PyMYB10. PyMYB10.1 is phylogenetically close to the known anthocyanin MYBI type regulators, particularly those from *Rosaceae*, but is more distantly related to the *Arabidopsis* AtTT2 [32], which controls proanthocyanidin synthesis; *Arabidopsis* AtMYB11 and AtMYB12 [33], which control flavonol synthesis; and FaMYB1 from strawberries [15], which represses anthocyanin synthesis (Fig 2).These results suggest that PyMYB10.1 plays a role in regulating anthocyanin synthesis.

### *PyMYB10*, *PyMYB10*.*1*and *PybHLH* gene expression analysis

**Fig 3:**
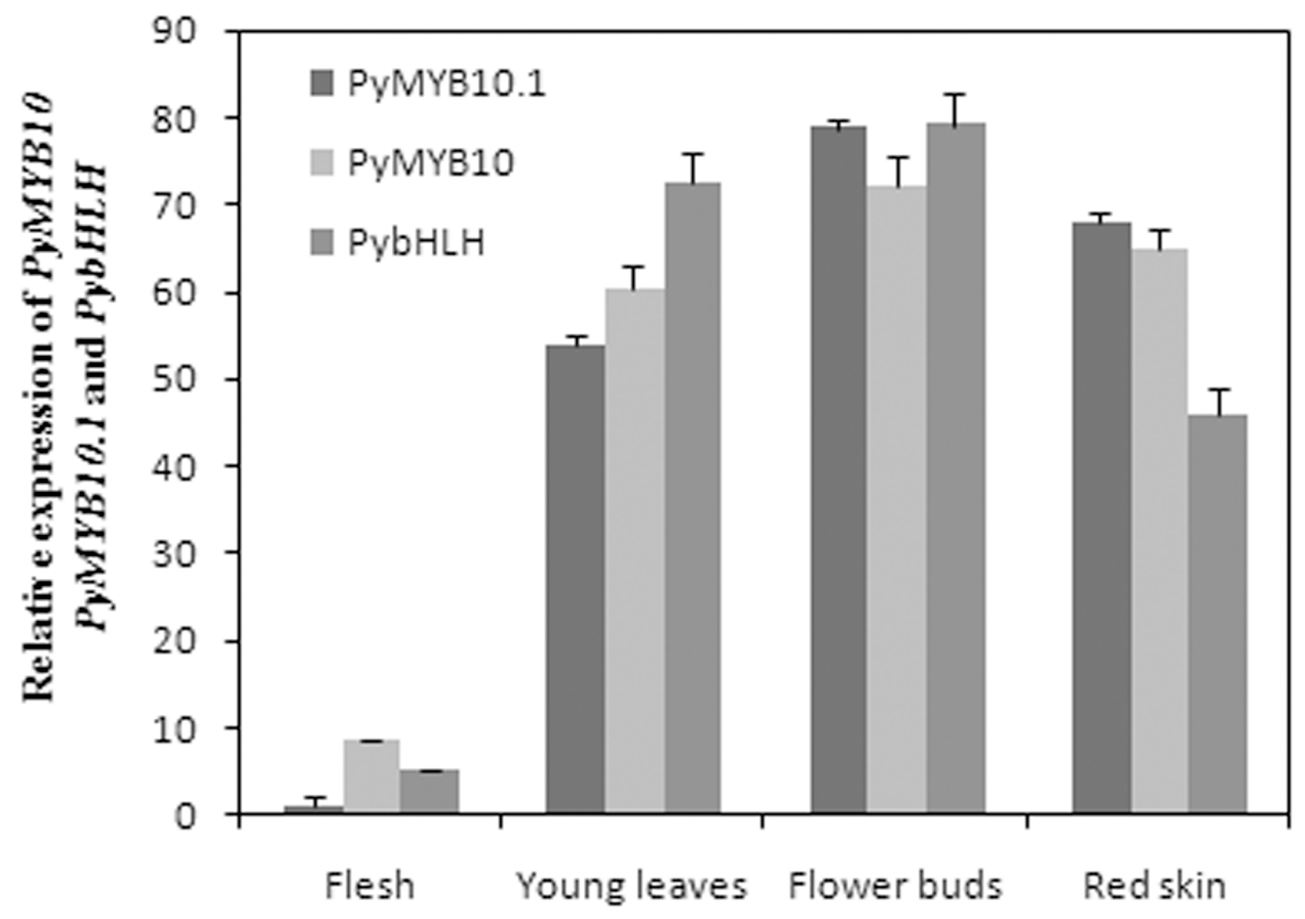
qPCR analysis of *PyMYB10*, *PyMYB10*.*1* and *PybHLH* in different tissues of ‘Aoguan’ pears including young leaves, flower buds, red pericarps, and flesh. *PyActin* was used as an internal control to normalize gene expression under identical conditions. Error bars represent means ± SE (n = 3).

To determine the spatial expression patterns of *PyMYB10* and *PyMYB10*.*1* in ‘Aoguan’, the transcripts of both genes in young leaves, flower buds, skin and flesh was profiled. Both *PyMYB10* and *PyMYB10*.*1* mRNAs were detected in all four tissues, and higher levels were found in the anthocyanin-rich red tissues of young leaves, fruit skins and flower buds. Only extremely low levels of *PyMYB10* and *PyMYB10*.*1* were observed in non-red fruit flesh. Similarly to *PyMYB10*and *PyMYB10*.*1*, *PybHLH* was predominantly expressed in the anthocyanin-rich red tissues (Fig 3).

**Fig 4:**
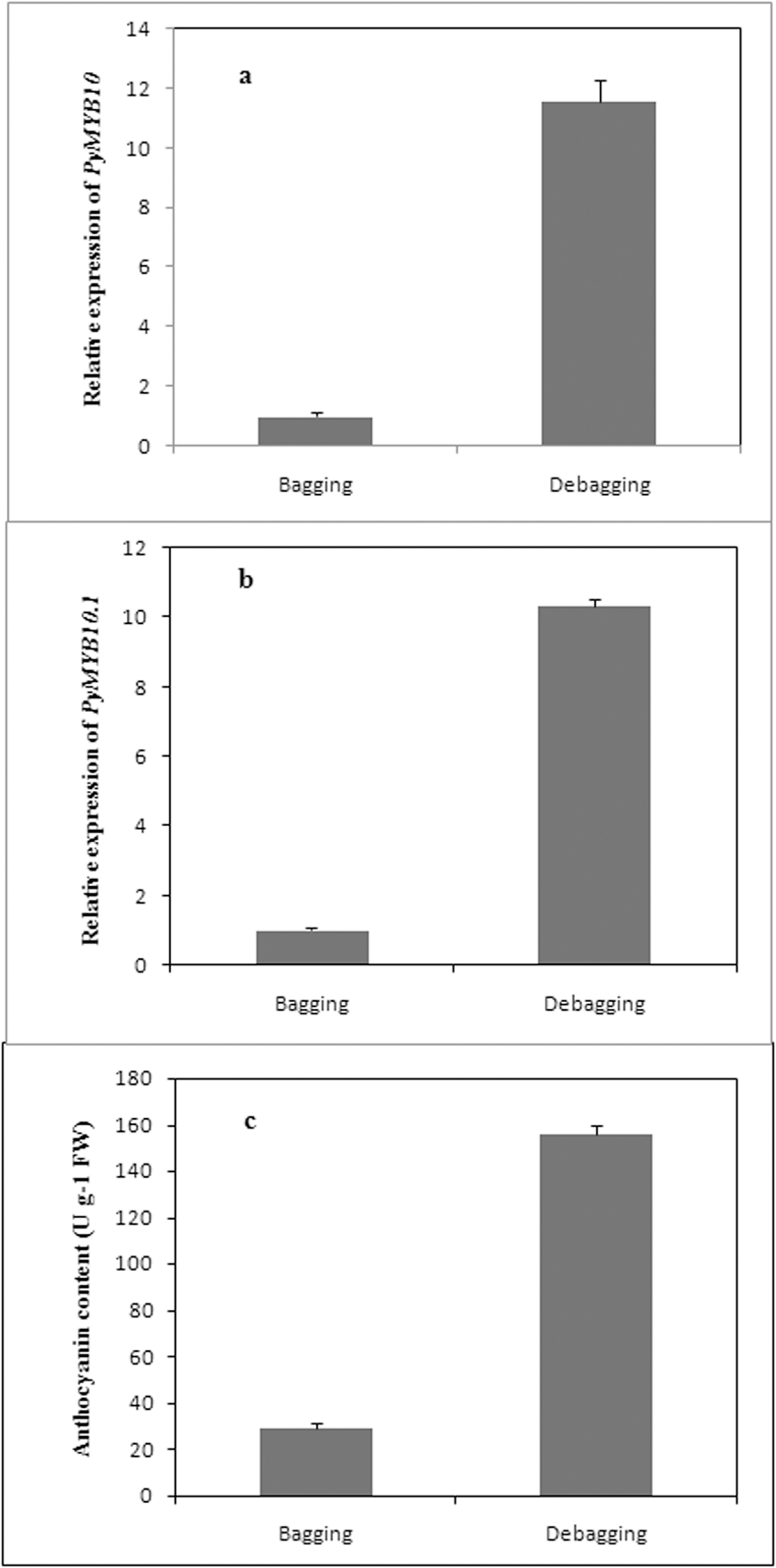
Anthocyanin accumulation and expression analysis of *PyMYB10* and *PyMYB10*.*1* in ‘Aoguan’ pears in response to light. (a) Expression analysis of *PyMYB10* in ‘Aoguan’ pears during bagging and debagging treatments. (b) Expression analysis of *PyMYB10*.*1* in ‘Aoguan’ pears during bagging and debagging treatments. (c) Anthocyanin contents in ‘Aoguan’ pears during bagging and debagging treatments. Fruits were collected 6 days after debagging. The fruits retained in the bags were sampled as controls. Error bars represent means ± SE (n = 3).

The expression levels of *PyMYB10* and *PyMYB10*.*1* in response to light treatment are shown in Fig 4. Compared to the bagged fruits, *PyMYB10* and *PyMYB10*.*1* were obviously upregulated in debagged fruit. The levels of the two genes were more than 11-foldhigher than in bagged fruit. Accordingly, the debagged fruits anthocyanin accumulation was obviously higher than bagged fruit (Fig 4).

### Protein–protein interactions of PyMYB10, PyMYB10.1 and PybHLHs

**Fig 5:**
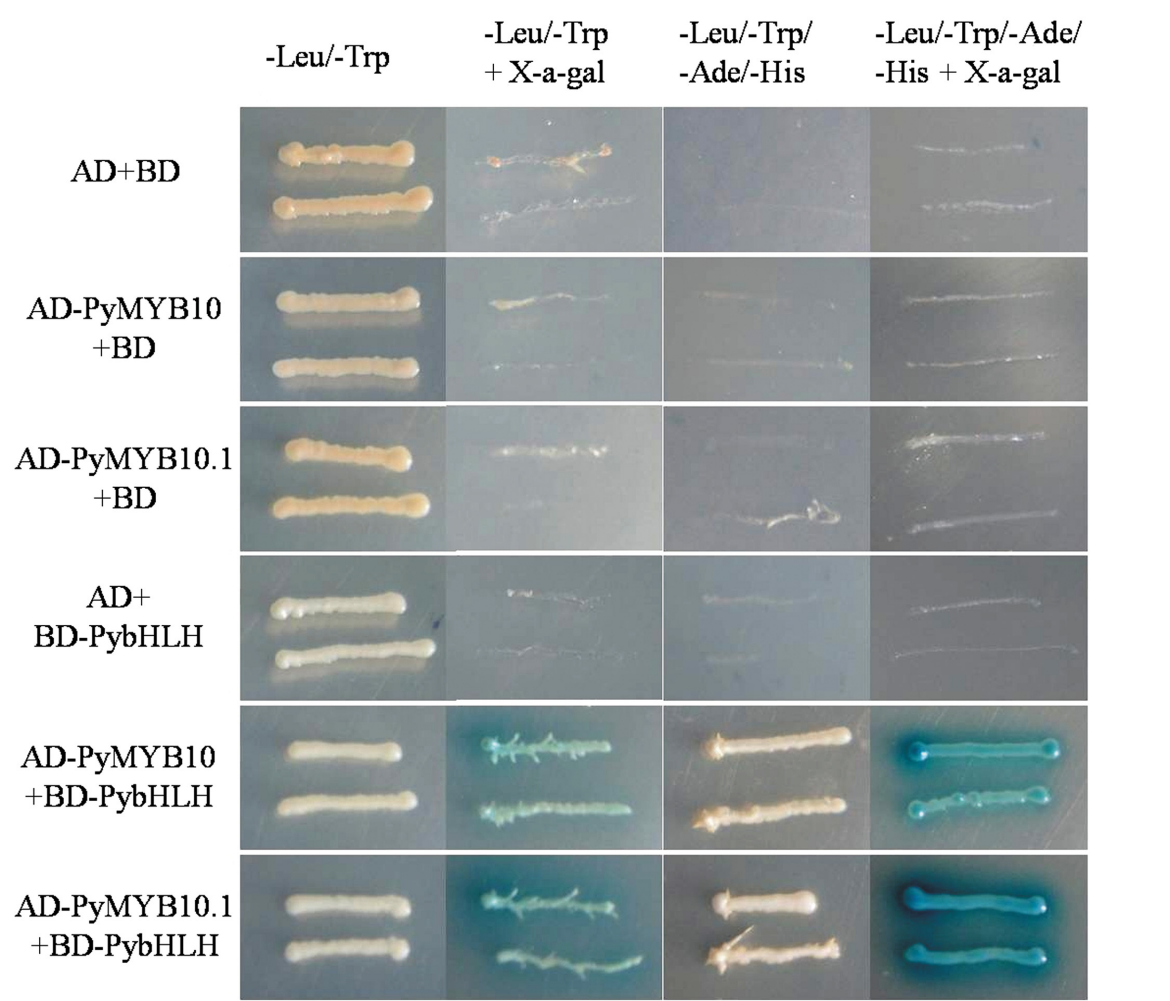
Interactions between PyMYB10 or PyMYB10.1 and PybHLH were detected through the yeast two-hybrid assay. AH109 yeast cells containing plasmids AD+BD, AD-PyMYB10+BD, AD-PyMYB10.1+BD, AD+BD-PybHLH, AD-PyMYB10+BD-PybHLH, or AD-PyMYB10.1+BD-PybHLH were grown on double- and quadruple-selection media. The X-gal assay was performed to confirm positive interactions.

A motif ([DE]Lx_2_[RK]x_3_Lx_6_Lx_3_R) which is necessary for the interaction between MYB and bHLH proteins was found in the R3-MYB domains of PyMYB10 and PyMYB10.1 [34]. To investigate whetherPyMYB10 or PyMYB10.1interacts with the pear bHLH anthocyanin regulator PybHLH, we employed a GAL4-basedyeast two-hybrid assay. The autoactivation of PyMYB10 and PyMYB10.1 was investigated first. Yeast harboring pAD-GAL4 (AD) plus pBD-GAL4-PyMYB10 (BD-PyMYB10) or BD-PyMYB10.1 grew well on the quadruple-selection medium, while the negative control, which contained pBD-GAL4 (BD) and pAD-GAL4-PyMYB10 (AD-PyMYB10) or AD-PyMYB10.1, did not grow, indicating that PyMYB10and PyMYB10.1can auto-activate. Subsequently, AD-PyMYB10, AD-PyMYB10.1 and BD-PybHLH were introduced into yeast. As shown in Fig 5, yeast cells containing either the combination of PyMYB10and PybHLH or PyMYB10.1and PybHLH grew well on all synthetic dropout(SD) selective media. These Y2H results demonstrate that PyMYB10 and PyMYB10.1 can physically interact with PybHLH in vitro.

**Fig 6:**
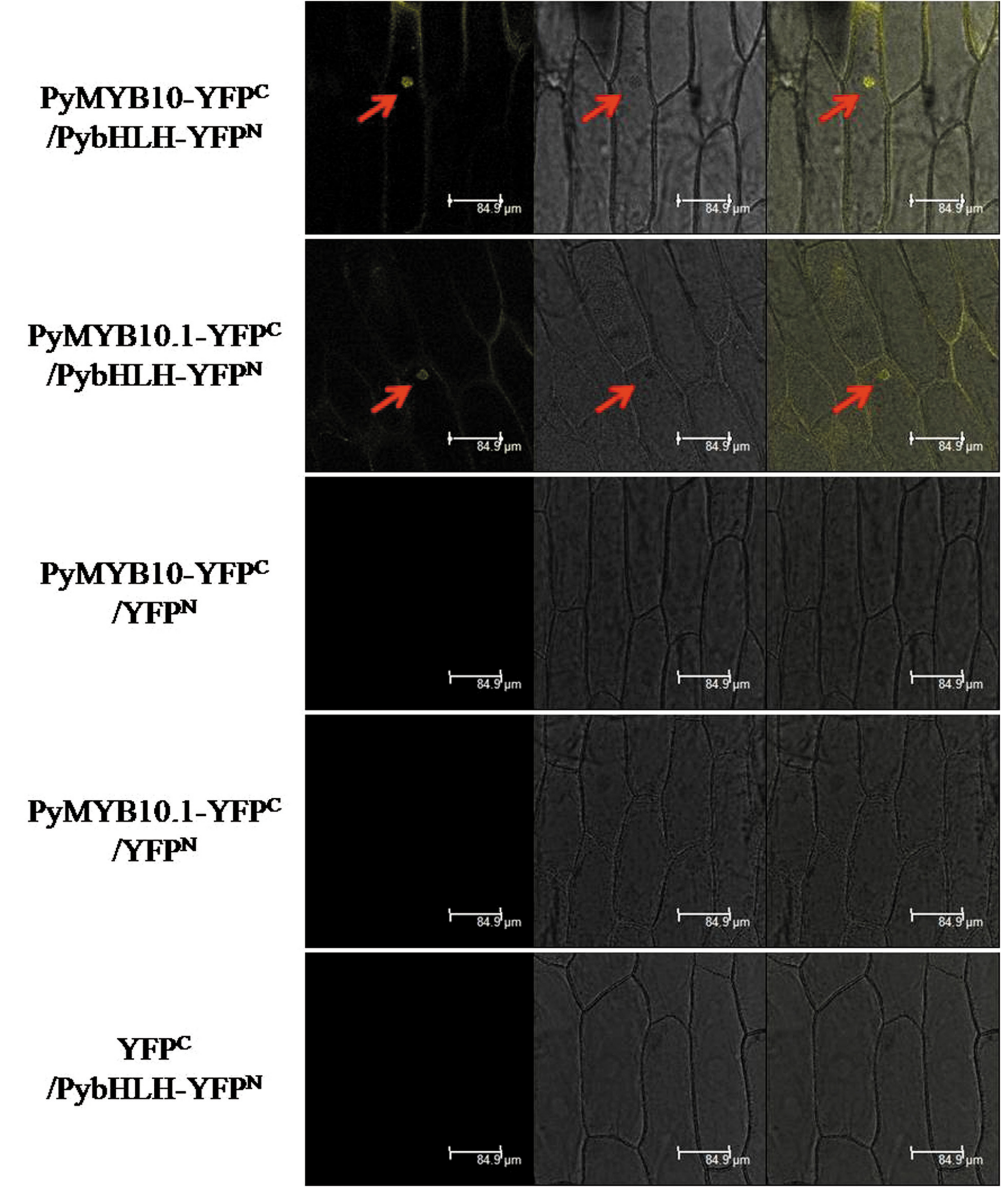
Interactions between PyMYB10 or PyMYB10.1 and PybHLH were detected using the BiFC assay. Onion epidermal cells were co-transfected with a mixture of *Agrobacterium* suspensions containing plasmids PyMYB10-YFPC + PybHLH-YFPN, PyMYB10.1-YFPC + PybHLH-YFPN, PyMYB10-YFPC + YFPN, PyMYB10.1-YFPC + YFPN, or YFPC + PybHLH-YFPN. YFP fluorescence signals were detected 48 h after transfection. Bar = 84.9 μm.

BiFC assays were further performed to investigate the interaction between PybHLH and PyMYB10or PyMYB10.1 in vivo. A plasmid harboring the N-terminal domain of YFP fused to the PyMYB10 (PyMYB10-NYFP) or PyMYB10.1 (PyMYB10.1-NYFP) cDNA and a plasmid containing the C-terminal domain of YFP fused to the PybHLH cDNA (PybHLH-CYFP) were transiently co-expressed in onion epidermis cells. Cells containing PyMYB10/PybHLH or PyMYB10.1/PybHLH were with a strong detectable fluorescence signal. Conversely, no fluorescence signal was detected in control cells (Fig 6).These BiFC results demonstrate that PyMYB10 and PyMYB10.1can interact with PybHLH in vivo.

### Transient luminescence assays of PyMYB10 and PyMYB10.1 activity

**Fig 7:**
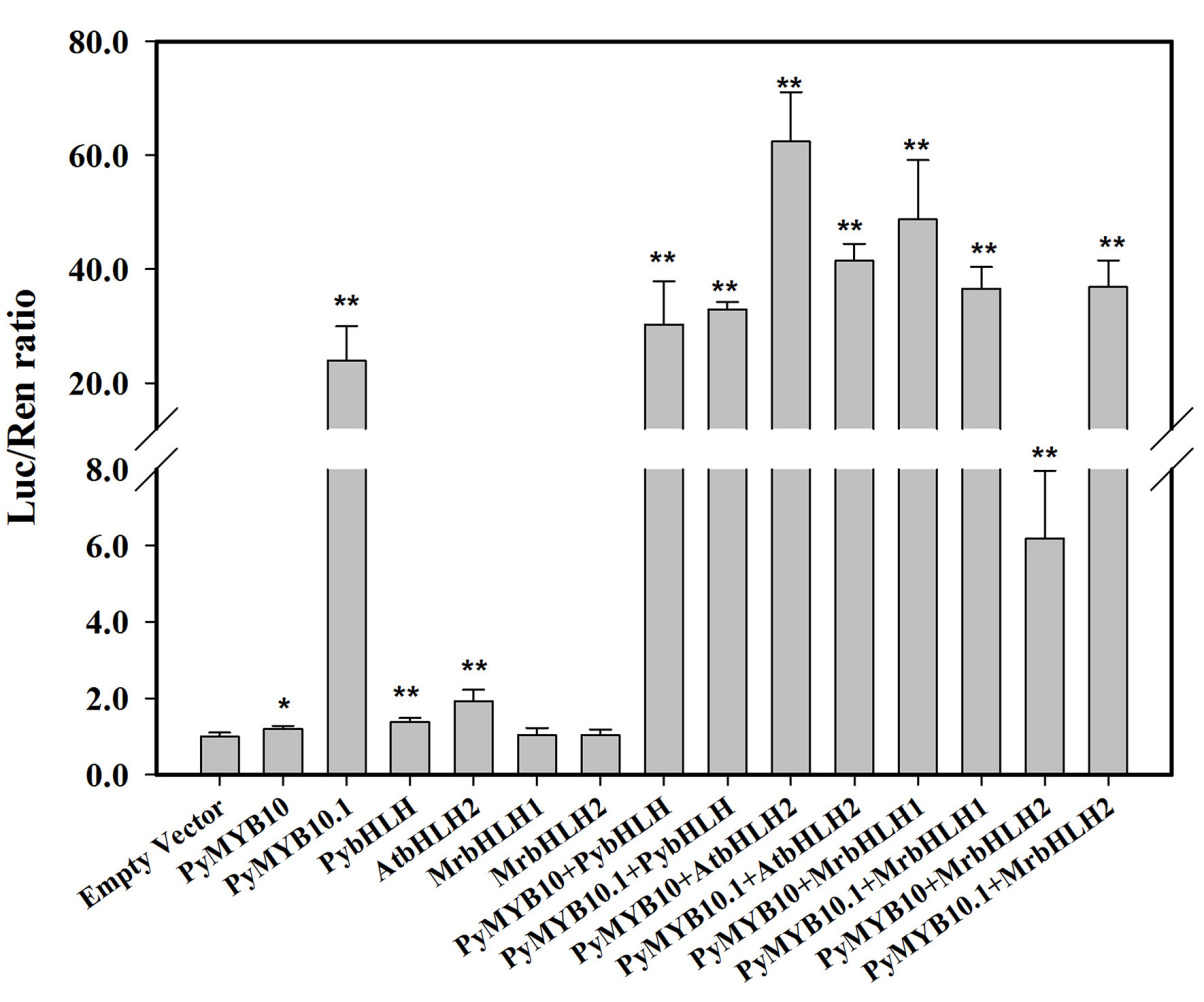
Activation of the *AtDFR* promoter by PyMYB10, PyMYB10.1 and anthocyanin-related bHLH transcription factors in a tobacco transient-expression assay. LUC and REN activities were analyzed three days after transformation. Error bars represent means ± SE (n = 6).Asterisks, * and **, indicate significant differences (P<0.05 and P<0.01).

To assess transient PyMYB10 and PyMYB10.1activity,a transient luminescence assay was performed.PyMYB10and PyMYB10.1were infiltrated into *N*. *benthamiana* leaves with the *AtDFR* promoter. As shown in Fig 7, both PyMYB10 and PyMYB10.1 could induce the *DFR* promoter alone. Compared to control, the induction by PyMYB10 was 1.2-fold higher. But the induction by PyMYB10.1 was more obvious, approximately 20-fold higher than that by PyMYB10. The increased activities of PyMYB10 and PyMYB10.1 were observed when they were with abHLH co-factor, and the highest activity was observed when they were co-infected with AtbHLH2. In all combinations tested, the induction by PyMYB10+AtbHLH2 was the highest.

**Fig 8:**
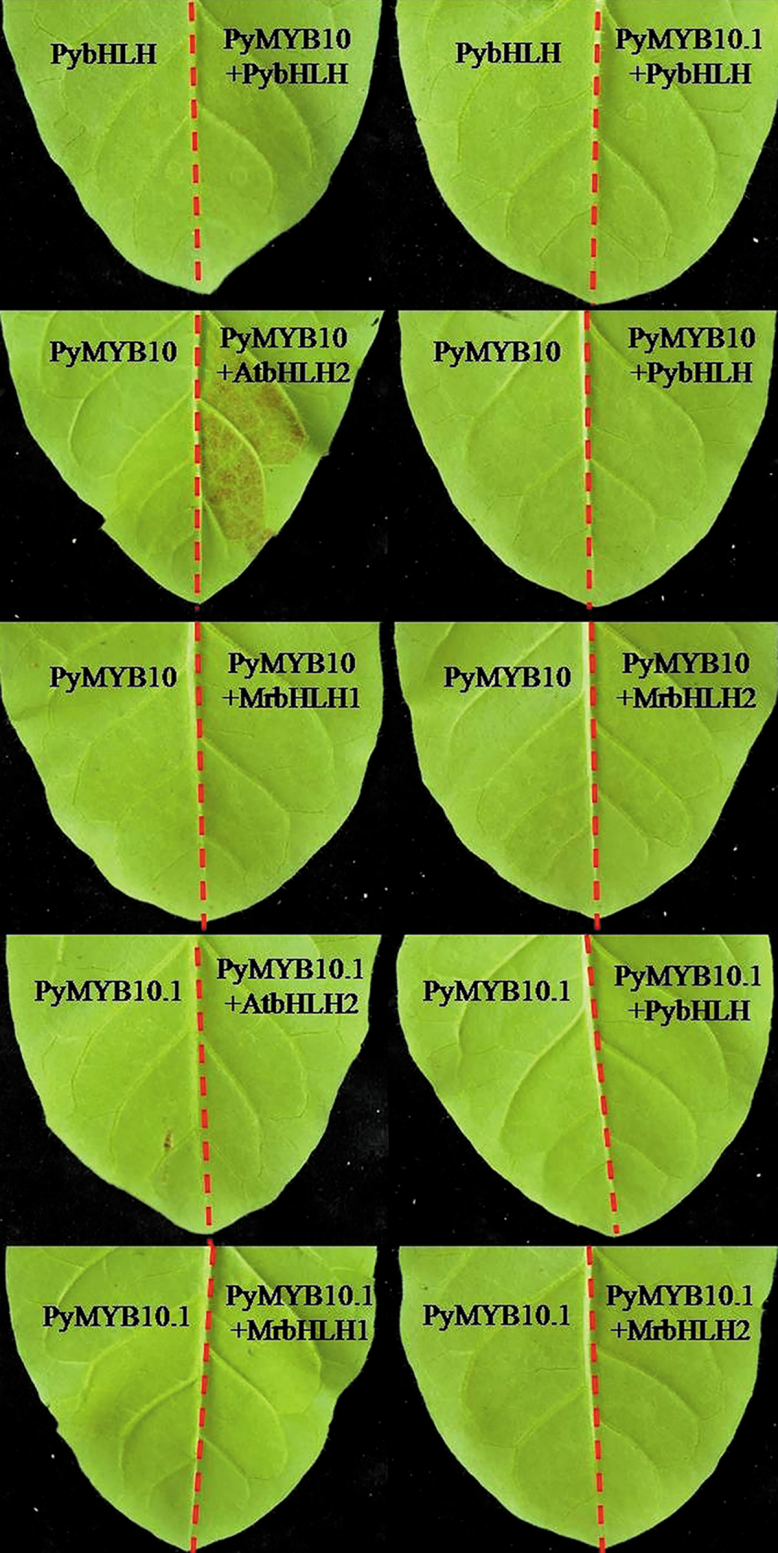
Patches of anthocyanin production in tobacco leaves infused with *Agrobacterium tumefaciens*. Photos of infiltration areas were taken 8 days after transformation with PyMYB10 or PyMYB10.1 together with AtbHLH2, PybHLH, MrbHLH1, or MrbHLH2.

Patches of foliar anthocyanin production can reportedly be induced by the co-expression of R2R3-MYBs and bHLHs in *Nicotiana tabacum* leaves [11].In the present study, the induction of anthocyanin biosynthesis by PyMYB10 and PyMYB10.1was tested, and only PyMYB10 could induce a patch of anthocyanin when co-expressed with AtbHLH2 (Fig 8).

## Discussion

Plant pigmentation is temporally and spatially regulated by the anthocyanin structural genes. The structural genes are primarily regulated at the transcriptional level. R2R3-MYBs play key roles in anthocyanin regulation through controlling the transcripts of anthocyanin structural genes. In recent years, numerous anthocyanin R2R3-MYB regulators in different fruit plants have been isolated and characterized. In the present study, a homologous of *PyMYB10*, *PyMYB10*.*1*, was cloned from ‘Aoguan’. A sequence analysis revealed that PyMYB10.1 shares a high sequence identity at R2R3-domain with anthocyanin-activating MYB transcription factors, particularly PyMYB10 from pears, but less homologous was detected at the full protein sequence. In a phylogenetic tree, PyMYB10.1 was closely related to anthocyanin-activating MYBI subgroup members, such as PyMYB10 [23], MdMYB10 [10] and FaMYB10 [35], suggesting that PyMYB10.1 may responsible for fruit anthocyanin regulation.

Previously, many R2R3-MYB TFs have been shown to display tissue-specific expression patterns that correlate strongly with anthocyanin accumulation. For example, the sweet potato *IbMYB1* was predominantly expressed in red tuberous roots [36]. Similarly, high transcript levels of *PyMYB10* and *PyMYB10*.*1* were detected in anthocyanin-rich flower buds, young leaves and fruit skins. In contrast, low levels of *PyMYB10* and *PyMYB10*.*1*transcripts were observed in the non-red fruit cortex. Therefore, the anthocyanin content must closely correlate with the *PyMYB10* and *PyMYB10*.*1*transcript levels in a tissue-specific manner. Furthermore, the higher transcript levels of *PybHLH* in anthocyanin-rich tissues were also observed. It seems that both *PyMYBs* and *PybHLHs* act roles in the regulation of anthocyanin synthesis, like in other plants.

Environmental factors, such as temperature and light, have been suggested to regulate anthocyanin biosynthesis via the up- or down-regulation of these R2R3-MYB transcription factors. In the present study, we found that the transcripts of *PyMYB10* and *PyMYB10*.*1*in pear skins were all up-regulated by sunlight, which is consistent with the expression of*MdMYB1* in apples [27] and *MrMYB1* in Chinese bayberries [11]. Sunlight likely regulates pear anthocyanin synthesis by up-regulating the expression of *PyMYB10* and *PyMYB10*.*1*.These results suggest that *PyMYB10* and *PyMYB10*.*1* likely act as anthocyanin activators in pears.

R2R3-MYBs regulate anthocyanin biosynthesis via activating the anthocyanin structural genes. Generally, the efficient induction of structural genes by R2R3-MYBs depends on the co-expression of a bHLH transcription factor, which jointly regulates target promoters. In apples, MdMYB10 could trans-activate the *AtDFR* promoter together with MdbHLH3 or MdbHLH33. The function of MdMYB10was weak in the absence of a bHLH co-factor[10].Similarly, the activity of the *AtDFR* promoter was higher when PyMYB10 and PyMYB10.1were co-expressed with a bHLH protein in tobacco than they were alone. Interestingly, the activity of the *AtDFR* promoter varied amongst the different combinations of R2R3-MYBs and bHLHs. For example, in apples, the combination of MdbHLH3 andMdMYB10resulted in a greater activation of *AtDFR* transcription compared with the combination of MdbHLH33and MdMYB10 [10]. As a result, these combinations led to different levels of anthocyanin. In the present study, the abilities of bHLH proteins with PyMYB10 or PyMYB10.1for the trans-activation of *AtDFR* depended on the species. In particular, the combination of PyMYB10 and AtbHLH2 showed trans-activation values more than ten-fold that of PyMYB10 and MrbHLH2. Clearly, the anthocyanin accumulation was detected in tobacco leaves co-infiltrated by the combination of PyMYB10 and AtbHLH2 but not by the combination of PyMYB10 and MrbHLH2. Therefore, the phenotypic differences may be partly attributed to the functional differences among these combinations. Surprisingly, the highly activation of *AtDFR* was detected in the combination of PyMYB10.1 and PybHLH, but none red pigmentation was induced. It has been reported that distinct anthocyanin structural genes may not be regulated by a single MYB protein or bHLH protein in several plants [37]. So, we speculated that the key structural genes that could not be regulated by PyMYB10.1 and PybHLH may exist. The anthocyanin structural genes should be further classified by experimental assays. Moreover, anthocyanin repressors may compete with anthocyanin activators [38]. Therefore, whether the negative regulators affect the anthocyanin accumulation process need to be clarified.

R2R3-MYBs are known to interact with bHLHs to control anthocyanin biosynthesis. In *Arabidopsis*, PAP1 could interact withEGL3. The motif ([DE]Lx_2_[RK]x_3_Lx_6_Lx_3_R) in the PAP1 R3-MYB domain is necessary for this interaction[17]. Similar results have shown that anthocyanin-related R2R3-MYBs containing this motif can interact with other heterologous EGL3-like bHLH proteins, such as MdMYB10 and MdbHLH3 from apples [10], VvMYC1 [39] and VvMYB5b from grapes [40], and BoMYB1 and BobHLH1 from purple cauliflower [41].In contrast, mutants of this motif abrogate the binding activity ofpurple cauliflowerBoMYB3 [41] and grape VvMYB5b [40] to BobHLH1 and VvMYC1, respectively. In the present study, a signature binding motif between MYB and bHLH proteins was also detected in both PyMYB10 and PyMYB10.1. Y2H and BiFC assays proved that both PyMYB10 and PyMYB10.1 interactwiththe recently identified pear bHLH TF PybHLH. Taking into account the expression patterns of *PyMYB10*, *PyMYB10*.*1* and *PybHLH*, the MYB-bHLH regulatory complexes may play important roles in pear anthocyanin accumulation.

More than one R2R3-MYB TF is often present in a single plant species and determines tissue-specific anthocyanin accumulation. However, some R2R3-MYB proteins abundantly regulate anthocyanin biosynthesis in the same organs. For example, the R2R3-MYB TFs*VvMYBA1* and *VvMYBA2* from grape both appear to control fruit skin coloration. Mutations of these two genes removed their ability in activating anthocyanin biosynthesis, and deactivating both genes results in a white cultivar [42].Deficient *AtPAP1* expression in *Arabidopsis* did not block the anthocyanin synthesis regulated by *AtPAP2*, *AtMYB113* and *AtMYB114* in vegetative tissues [13].In pears, we observed that both *PyMYB10* and *PyMYB10*.*1*arepredominantly expressed in anthocyanin-rich tissues and up-regulated by light. In addition, all of these R2R3-MYBs form protein complexes with bHLHs to regulate anthocyanin structural genes. These results indicate that *PyMYB10* and *PyMYB10*.*1* may play redundant roles in pear anthocyanin regulation, as similarly reported in grapes and *Arabidopsis*. However, the anthocyanin regulatory ability of each protein significantly differed in the presence of a bHLH co-factor. Understanding the anthocyanin-activating functions of PyMYB10 and PyMYB10.1 will promote the biological breeding of red sand pears.

## Supporting Information

S1 Table Primers used for real-time quantitative PCR. (DOCX)

S2 Table Primers used in the tobacco transient-expression assay. (DOCX)

S3 Table Primers used in the yeast two-hybrid (Y2H) assay. (DOCX)

S4 Table Primers used in the bimolecular fluorescence complementation (BiFC) assay. (DOCX)
