# Supplementary material for: PyMYB10 and PyMYB10.1 Interact with bHLH to Enhance Anthocyanin Accumulation in Pears
Source: PLoS One. 2015 Nov 4;10(11):e0142112. doi: 10.1371/journal.pone.0142112 (PMC4633228; doi:10.1371/journal.pone.0142112)
Supplement: S1 Table — (DOCX) [file pone.0142112.s001.docx]

**S1 Table. Primers used for real-time quantitative PCR.**

| Gene | Primer | Sequence (5’ 3’) |
| --- | --- | --- |
| *PyMYB10* | Forward | CAGCAGAAGATTTAAGTACGCCATC |
|  | Reverse | TTCTAACAAGGTCTCCCACCAATC |
| *PyMYB10.1* | Forward | AACCACGGAGGGCTAGAGTT |
|  | Reverse | GCCTACCATTGTCGATTGTGC |
| *PybHLH* | Forward | AGCTGGTATCGGAGGACCTT |
|  | Reverse | ATCGTCTGCTTTGTGAGGGG |
| *PyActin* | Forward | TCCAGAAGAGCATCCAGTCC |
|  | Reverse | GCCAGGTCCAAACGAAGG |
