# Supplementary material for: PyMYB10 and PyMYB10.1 Interact with bHLH to Enhance Anthocyanin Accumulation in Pears
Source: PLoS One. 2015 Nov 4;10(11):e0142112. doi: 10.1371/journal.pone.0142112 (PMC4633228; doi:10.1371/journal.pone.0142112)
Supplement: S2 Table — (DOCX) [file pone.0142112.s002.docx]

**S2 Table. Primers used in the tobacco transient-expression assay.**

| Gene | Primer | Sequence (5’ 3’) |
| --- | --- | --- |
| *PyMYB10* | Forward | GCGAGCTCATGGAGGGATATAACGTTAAC (*Sac*I) |
|  | Reverse | GCGGTACCCTATTCTTCTTTTGAATGATTC (*Kpn*I) |
| *PyMYB10.1* | Forward | GCGAGCTCATGGAGGATAGTAATTTGC (*Sac*I) |
|  | Reverse | GCCTCGAGCTAAATCTTAGTTATCTCTTC (*Xho*I) |
| *PybHLH* | Forward | GCGAGCTCATGGCTCAGAATCATGAG (*Sac*I) |
|  | Reverse | GCCTCGAGTCAGCACTTACCAGCAATTTTCC (*Xho*I) |
| *AtbHLH2* | Forward | ATGAGCTCATGGCAACCGGAGAAAACAG (*Sac*I) |
|  | Reverse | GCGGTACCTTAACATATCCATGCAACCC (*Kpn*I) |
| *MrbHLH1* | Forward | TAGAATTCATGGCTGCACCGCCGAGTAGC(*EcoR*I) |
|  | Reverse | CGAAGCTTCTACGAGTCATTGTGGGGTAT(*Hind*III) |
| *MrbHLH2* | Forward | CGGGATCCATGGCCAATGGCACTCAAAC(*BamH*I) |
|  | Reverse | TAGTCGACTCAACACCTGCAAGCGATTTTC(*Sal*I) |
