# Supplementary material for: PyMYB10 and PyMYB10.1 Interact with bHLH to Enhance Anthocyanin Accumulation in Pears
Source: PLoS One. 2015 Nov 4;10(11):e0142112. doi: 10.1371/journal.pone.0142112 (PMC4633228; doi:10.1371/journal.pone.0142112)
Supplement: S3 Table — (DOCX) [file pone.0142112.s003.docx]

**S3 Table. Primers used in the yeast two-hybrid (Y2H) assay.**

| Gene | Primer | Sequence(5’ 3’) |
| --- | --- | --- |
| *PyMYB10.1* | Forward | TCCATATGATGGAGGATAGTAATTTGCTGG (*Nde*I) |
|  | Reverse | CGGGATCCAATCTTAGTTATCTCTTCTTC (*BamH*I) |
| *PyMYB10* | Forward | TCCATATGATGGAGGGATATAACGTTAACTTGAG (*Nde*I) |
|  | Reverse | CGGGATCCTTCTTCTTTTGAATGATTCCAAAG (*BamH*I) |
| *PybHLH* | Forward | ATCCCGGGTATGGCTCAGAATCATGAGAGG (*Xma*I) |
|  | Reverse | AACTGCAGGCACTTACCAGCAATTTTCC (*Pst*I) |
