# Supplementary material for: PyMYB10 and PyMYB10.1 Interact with bHLH to Enhance Anthocyanin Accumulation in Pears
Source: PLoS One. 2015 Nov 4;10(11):e0142112. doi: 10.1371/journal.pone.0142112 (PMC4633228; doi:10.1371/journal.pone.0142112)
Supplement: S4 Table — (DOCX) [file pone.0142112.s004.docx]

**S4 Table. Primers used in the bimolecular fluorescence complementation (BiFC) assay.**

| Gene | Primer | Sequence (5’ 3’) |
| --- | --- | --- |
| *PyMYB10.1* | Forward | CGGGATCCATGGAGGATAGTAATTTGCTGG (*BamH*I) |
|  | Reverse | GCGTCGACAATCTTAGTTATCTCTTCTTC (*Sal*I) |
| *PyMYB10* | Forward | CGGGATCCATGGAGGGATATAACGTTAACTTGAG (*BamH*I) |
|  | Reverse | GCGTCGACTTCTTCTTTTGAATGATTCCAAAG (*Sal*I) |
| *PybHLH* | Forward | CGGGATCCATGGCTCAGAATCATGAGAGG (*BamH*I) |
|  | Reverse | GCGTCGACGCACTTACCAGCAATTTTCC (*Sal*I) |
